# Uncovering human kinase substrates in nipah proteome

**DOI:** 10.3389/fbinf.2025.1678189

**Published:** 2025-12-05

**Authors:** Vineetha Shaji, Akash Anil, Ayisha A. Jabbar, Althaf Mahin, Ahmad Rafi, Amjesh Revikumar, Sowmya Soman, Ganesh Prasad, Sneha M. Pinto, Yashwanth Subbannayya, Abhithaj Jayanandan, Rajesh Raju

**Affiliations:** 1 Centre for Integrative Omics Data Science (CIODS), Yenepoya (Deemed to be University), Mangalore, India; 2 Center for Systems Biology and Molecular Medicine (CSBMM), Yenepoya Research Centre, Yenepoya (Deemed to Be University), Mangalore, India; 3 Department of Biochemistry, Yenepoya Medical College, Yenepoya (Deemed to be University), Mangalore, India; 4 School of Biosciences, Faculty of Health and Medical Sciences, University of Surrey, Guildford, United Kingdom; 5 Surrey Institute for People- Centred AI, University of Surrey, Guildford, United Kingdom

**Keywords:** Nipah virus, phosphorylation, computational prediction tools, host–virus interactions, kinase-substrate phosphomotif search, protein–protein docking, molecular dynamics simulation

## Abstract

Nipah virus (NiV) is a zoonotic pathogen that causes recurrent outbreaks with considerable implications for public health. Viruses engage host kinases to phosphorylate viral proteins, aiding replication and host disruption. Identifying NiV phosphoproteins and their host kinases is therefore critical for understanding the mechanism of infection and developing therapeutics. We performed kinase-substrate phosphomotif analysis based on prior studies and employed computational tools to identify putative phosphosites in NiV proteins and corresponding host kinases. Redundancy analysis highlighted key kinases capable of phosphorylating multiple NiV proteins and high-potential viral substrates. Integration with human–viral protein–protein interaction data revealed human kinase substrate proteins in human that interact with NiV proteins, while conservation analysis assessed phosphosites across nine NiV proteins in various strains. The functional significance of the identified and predicted viral substrates and their corresponding host kinases was further validated through *in silico* docking and molecular dynamics simulation (MD). Motif-based kinase-substrate analysis identified 51 human kinases predicted to target 1180 phosphorylation sites across nine NiV proteins, including key human kinases such as Eukaryotic elongation factor 2 kinase [EEF2K], Haploid germ cell-specific nuclear protein kinase [HASPIN], Mitogen-activated protein kinase 9 [MAPK9], Microtubule-associated serine/threonine-protein kinase 2 [MAST2], and Spleen tyrosine kinase [SYK], with the potential to phosphorylate multiple sites across NiV proteins. Using computational prediction tools, we identified several potential phosphorylation sites on NiV proteins, along with their corresponding candidate human kinases. *In silico* docking revealed interactions between EEF2K and both the NiV Fusion Glycoprotein and NiV Phosphoprotein (P), MAPK9 with the NiV Matrix Protein, and HASPIN with NiV RNA-dependent RNA polymerase. MD simulations of the EEF2K–NiV Fusion Glycoprotein complex confirmed the stability of this interaction. Leucine-rich repeat serine/threonine-protein kinase 2 [LRRK2], HASPIN, MAST2, and EEF2K were the human kinases predicted to phosphorylate experimentally validated sites on NiV nucleocapsid (N), P, and W proteins. Furthermore, through an extensive literature review, we investigated the therapeutic potential of targeting these kinases using known inhibitors and identified compounds that could potentially be repurposed as antiviral agents against NiV infection. Our findings indicate that EEF2K phosphorylates key NiV proteins at conserved phosphosites across variants, underscoring the pathogenic significance of kinases in NiV infection and their potential as therapeutic targets.

## 1 Introduction

Nipah Virus (NiV) is an emerging *Paramyxovirus* (genus *Henipavirus*, subfamily *Paramyxovirinae*, family *Paramyxoviridae*, order *Mononegavirales*) that can cause severe respiratory illness and fatal encephalitis in humans. It is a negative-sense, single-stranded, non-segmented, enveloped RNA (ssRNA) virus with helical symmetry (Singh et al., 2019). The genome of NiV is 18.2-kb (Ranadheera et al., 2018) and encodes six structural proteins including nucleocapsid (N), phosphoprotein (P), matrix (M), fusion (F), attachment (G), and RNA-dependent RNA polymerase (L) as well as the nonstructural proteins like V, W, and C, in the order 3′-N-P/V/W/C-M-F-G-L-5′ (Omi-Furutani et al., 2010; Martinez-Gil et al., 2017). The P, V, and W proteins play critical roles in the replication and virulence of NiV (Keiffer et al., 2020). Between late 1998 and mid-1999, NiV triggered a severe encephalitis outbreak among pig-contact individuals in Malaysia and Singapore, evidencing zoonotic spillover (Chattu et al., 2018). Each viral structural protein plays a critical role in various stages of the viral life cycle. In particular, the viral core or nucleocapsid, composed of the N, P, and L proteins, encompasses all factors essential for viral transcription and genome replication, ensuring virus can effectively hijack the host’s cellular machinery to produce new viral particles and perpetuate its infectious cycle. The Malaysian outbreak reported 276 cases with a 38% fatality rate, while later outbreaks in India and Bangladesh showed higher lethality, ranging from 43% to 100% (Chattu et al., 2018). Sporadic NiV outbreaks have since emerged in South-East and South Asia, notably in Bangladesh, India, and the Philippines (Verma et al., 2024). The most recent NiV outbreak occurred in Kozhikode, Kerala, India, between August 30 and 11 September 2023, with the strain matching the Bangladesh variant (Verma et al., 2024).

NiV is classified as a biosafety level-4 pathogen and select agent, necessitating research in BSL-4 laboratories (Epstein et al., 2006; Singh et al., 2019). The most frequent routes of disease transmission are ingestion of bat-contaminated date palm sap or contact with diseased intermediate hosts (Khan et al., 2024). Bat-to-human transmission is the most prevalent mode, with *Pteropus* bats serving as the natural reservoir, while intermediate hosts are also implicated (Halpin et al., 2011; Hauser et al., 2021). NiV typically manifests in clusters and produces encephalitis or acute respiratory distress after an incubation period of 5–14 days. Early symptoms including fever and headache develop into confusion or coma within 24–48 h (Rahim et al., 2024; Islam et al., 2023). Early symptoms include dizziness, vomiting, muscle soreness, and diarrhea, but neurological symptoms such as myoclonus, cerebellar dysfunction, and reduced consciousness (55 percent in Malaysia) suggest brainstem involvement (Banerjee et al., 2019; Ching et al., 2015). Due to its high fatality rate, rapid progression, and the lack of affordable diagnostics, research, vaccinations, or cures, effective diagnosis and therapy development for the deadly NiV remain hindered (Shariff and Shariff, 2019).

Previous studies have indicated that, despite differences in the locations of phosphorylation sites, the kinases responsible for protein phosphorylation show a certain degree of uniformity across *paramyxoviruses* (Shiell et al., 2003). Furthermore, LC-MS/MS (Liquid Chromatography-Mass Spectrometry/Mass Spectrometry) analysis using an ESI-IT-MS instrument has been utilized to examine the phosphoproteomes of newly identified *paramyxoviruses*, HeV and NiV, offering significant information regarding their post-translational modifications (Shiell et al., 2003). Huang et al. (2011), discovered a quick turnover of phosphorylation in the NiV-N and identified phosphorylated S451 via mass spectrometry when cellular phosphatases were inhibited, underscoring its essential function in viral transcription and replication (Huang et al., 2011). Edwards et al. (2020) revealed that the residues T410, T420, T438, and S449 within the W protein of NiV are conserved across both NiV strains and the W protein of Hendra virus (HeV). Interestingly, the conserved serine residue known as S449 is essential for facilitating phosphorylation-dependent interactions between the W proteins of both viruses and each of the seven 14-3-3 protein family isoforms. The W protein’s C-terminal region is where this binding preference occurs (Edwards et al., 2020). The viral post-translational modification database (VPTMdb), an online resource for viral and host PTM (Post-Translational Modifications) sites contains a limited amount of information on the phosphorylation events linked to the NiV (Xiang et al., 2021). However, the phosphorylation patterns of host protein kinases during NiV infection are poorly understood due to the relatively low number of phosphoproteomic studies. Unlike previous studies such as Huang et al. (2011) and Edwards et al. (2020), which focused on experimentally identifying specific phosphorylation sites within individual NiV proteins, our study provides the first systematic, *in silico* kinase-substrate motif analysis to map potential host kinase interactions across the entire NiV proteome.

Phosphorylation plays a critical role in viral infection, replication, and the cytotoxic impact on host cells, positioning it as a significant focus in antiviral research (Shaji et al., 2025; Keating and Striker, 2012). A number of FDA-approved tyrosine kinase inhibitors, which were initially designed for cancer therapy, are currently undergoing testing in animal models and clinical trials to assess their efficacy in targeting phosphorylation for the treatment of viral infections (Keating and Striker, 2012). The current study utilizes a kinase-substrate phosphomotif pattern analysis to discover human kinases associated with NiV proteins. Further, we utilize computational tools and available datasets to identify phosphorylation sites and their interactions with host kinases. Finally, using *in silico* analysis, we explore the relationships between these viral phosphosites and kinase activation in the context of NiV infection. This research could open up novel therapeutic strategies for NiV infections and laying the foundation for future One health-based investigations into other viral infections through a better understanding of the interactions between host protein kinases and viral proteins.

## 2 Materials and methods

### 2.1 Phospho-kinase motif selection

We utilized kinase substrate sequence motifs of human kinases from previous studies by Sugiyama et al. and Poll et al. (2024) to identify potential phosphorylation sites in NiV proteins (Poll et al., 2024; Sugiyama et al., 2019). Sugiyama et al. (2019) identified 175,574 potential direct substrates, and phosphorylation patterns of 385 recombinant human protein kinases were utilized to derive kinase-substrate sequence preference motifs (Sugiyama et al., 2019). Expanding on this research, Poll et al. (2024) established a comprehensive set of kinase substrate sequence preference motifs, and visual representations of 13-amino-acid-centered substrate preferences for each kinase were generated using PTM-Logo software (Poll et al., 2024). Kinase logos generated using a minimum of 30 target amino acid sequences were utilized.

From the analysis, out of 384 human kinase substrate sequence motif logos, 330 statistically significant kinase substrate motifs were selected for further analysis. A total of 52 kinases were excluded based on specific criteria: For the seven kinases omitted in Poll et al. (2024), low-intensity residues mean that the motif patterns were generated from insufficient or noisy data, making the individual residues difficult or impossible to reliably read or identify. 42 kinases that exhibited fewer than 30 target sequences without consensus residues were removed, and three kinases with no statistically significant residues were also excluded.

### 2.2 Integrated computational and kinase substrate motif-based approach for predicting phosphorylation sites in NiV proteins and identifying upstream kinases

We analyzed nine protein sequences retrieved from UniProt and NCBI databases (https://www.uniprot.org/ and https://www.ncbi.nlm.nih.gov/) using an in-house Python script to predict potential phosphorylation sites in NiV proteins. Multiple sequence alignment using Clustal Omega confirmed that these sequences are highly conserved with minimal variation. Three computational tools were employed: NetPhos 3.1 (Blom et al., 1999) for kinase-specific site prediction, GasPhos (Chen et al., 2020) for machine learning-based kinase-substrate prediction, and GPS 6.0 (Chen et al., 2023) for identifying kinase-specific phosphorylation sites. These predictions were carried out to anticipate potential phosphorylation sites within NiV proteins. Furthermore, we compared the predicted kinases and their associated potential phosphorylation sites from these tools with results obtained through motif-based kinase analysis to identify overlapping predictions and assess kinase-specific phosphorylation patterns.

The complete NiV protein FASTA sequences were retrieved from the UniProt database (taxonomy ID: 3052225). The human kinase-specific substrate phosphomotif sequences were searched against FASTA sequences of nine NiV proteins to identify potential signature sequences in virus substrates. The UniProt database’s protein sequences were aligned using Clustal Omega (Li, 2003) to investigate the similarities or differences in viral phosphosite motifs among various strains and isolates. The conservation of these motifs was assessed by analyzing the amino acid sequences at each position (−5 to +5) to pinpoint matches of phosphomotifs within the sequences. These tools are used for identifying and predicting candidate phosphorylation sites and their corresponding sites in viral proteins. Notably, their predictions are primarily based on consensus sequence motifs shared within kinase families. As such, they typically predict potential kinases at the family or subfamily level, rather than pinpointing the exact individual kinase responsible for phosphorylation of these NiV proteins. To enhance the accuracy of kinase prediction, we integrated results from computational tools with motif-based analysis, allowing us to identify overlapping predictions and gain deeper insights into kinase-specific phosphorylation patterns in NiV proteins.

### 2.3 Exploration of human kinase interactions with NiV proteins through a literature-based approach

In order to determine if previously identified phosphosites of NiV protein have been reported in existing literature, a thorough evidence-based literature search was performed. Experimentally confirmed phosphorylation sites for both kinases and NiV proteins were gathered from multiple sources, including phosphorylation databases like VPTMdb (Xiang et al., 2021) and various research articles (Shiell et al., 2003; Huang et al., 2011; Edwards et al., 2020).

### 2.4 Structural validation of predicted human kinase-viral protein interactions via docking studies

The kinase-substrate phosphomotif pattern analysis identified several human kinases that could potentially phosphorylate various NiV proteins, including RNA-Directed RNA Polymerase L, Non-Structural Protein V, Fusion Glycoprotein F0, Nucleoprotein, Glycoprotein, Phosphoprotein, Matrix Protein, Protein W, and Protein C. Furthermore, the protein–protein docking analysis was carried out to examine these interactions and demonstrated that several of the predicted phosphorylation sites on NiV proteins formed strong and specific interactions with human kinases. Docking analyses were conducted only on regions of the kinases and NiV proteins for which high-quality PDB structures were available, while any unresolved or incomplete regions were excluded from the study. The selected structures represented the models with the maximum sequence coverage, ensuring inclusion of the residues of interest within the resolved regions.

NiV protein structures were retrieved from the RCSB Protein Data Bank (RCSB PDB). These included fusion glycoprotein f0 (PDB ID: 5EVM, with a resolution of 3.37 Å) (Xu et al., 2015), Glycoprotein (PDB ID: 2VWD, with a resolution of 2.25 Å) (Bowden et al., 2008), Nucleocapsid (PDB ID: 7NT5, with a resolution of 3.50 Å (Ker et al., 2021), Matrix protein (PDB ID: 7SKT, with a resolution of 2.05 Å (Norris et al., 2022) and Cryo-EM structure of the NiV polymerase (L) bound to the tetrameric phosphoprotein (P) (PDB ID: 9FUX, with a resolution of 2.49 Å) (Balikci et al., 2025). The Protein Data Bank (Berman et al., 2000) were utilized to obtain the 3D structure of human kinases EEF2K (PDB ID: 8FNY - Nucleotide-bound structure of a functional construct of eukaryotic elongation factor 2 kinase) with a resolution of 2.22 Å (Piserchio et al., 2023), HASPIN (PDB ID: 3DLZ - Crystal structure of human HASPIN in complex with AMP) with a resolution of 1.85 Å (Eswaran et al., 2009) and MAPK9 (PDB ID: 7N8T - Crystal Structure of AMP-bound Human JNK2) with a resolution of 1.69 Å (Lu et al., 2023) were selected. All protein structures were optimized and energy-minimized using the Protein Preparation Wizard with the OPLS4 force field in Schrödinger Maestro 2025–3 Physicochemical properties of the identified kinases were calculated using the MPP Profiler tool (Sganzerla Martinez et al., 2024).

Protein-protein docking was performed using BioLuminate (Navhaya et al., 2024) in Schrodinger Maestro 2025-3 to examine interactions between NiV proteins and human kinases. The NiV proteins Fusion Glycoprotein F0, Glycoprotein, Nucleocapsid, Phosphoprotein, Matrix Protein, and RNA-Directed RNA Polymerase L were selected as ligands, while human protein kinases EEF2K, HASPIN, MAPK9 served as receptors. The docking complex structure coordinates of EEF2K and the NiV fusion glycoprotein f0, along with the corresponding favorable residue interactions, were obtained using Schrödinger Maestro 2025–3 (Schrödinger, LLC, New York, NY, United States). Prior to docking, the protein structures were pre-processed using the Protein Preparation Wizard in Schrödinger Maestro 2025-3. The preparation steps included assigning correct bond orders, forming disulfide bonds, adjusting ionization states, removing unwanted water molecules, metals, and cofactors, correcting group orientations, capping termini, and adding any missing atoms or side chains. Partial charges were assigned, and hydrogen atoms were added, with all structures set to a standard protonation state at pH 7. Protein-protein docking was conducted by specifying the attraction and repulsion constraints based on the catalytic domain and phosphosite with respect to NiV proteins. The human kinase-NiV protein docking complex interactions were analyzed using the protein interaction analysis module in Biologics. Thirty poses were generated for the complex and ranked based on PIPER cluster size and PIPER pose energy, which evaluates receptor-ligand interactions and is efficiently computed using Fast Fourier Transforms (Mondol et al., 2023). From this top-scoring complex was selected for further analysis. We performed docking of the EEF2K–Fusion Glycoprotein F0 complex using the HADDOCK web server, which enables modeling of biomolecular complexes while incorporating experimental or predicted restraints (de Vries et al., 2010). Docking was carried out based on defined parameters, and the top 10 clusters generated by HADDOCK were analyzed. The top-ranked cluster was considered the most reliable, with its Z-score indicating the number of standard deviations from the average cluster score (more negative values indicate higher reliability) (de Vries et al., 2010).

### 2.5 Molecular dynamics simulation of the EEF2K–Fusion glycoprotein F0 complex

The coordinates of the docking complex structure between EEF2K and the NiV fusion glycoprotein f0, along with the favorable residue interactions, were obtained from the protein–protein docking results generated using BioLuminate in Schrödinger Maestro 2025-3 (Schrödinger, LLC, New York, NY, United States). These complexes were placed in orthorhombic boxes of size 10 Å × 10 Å × 10 Å and solvated with single point charge (SPC) water molecules using the Desmond System Builder (Schrödinger, LLC, New York, NY, United States). All simulation systems were neutralized with counterions, and a salt concentration of 0.15 M NaCl was maintained. Simulations were conducted for 300 ns using Desmond, in triplicate, with each run initiated by assigning a different set of initial velocities to the atoms (Antony and Vijayan, 2021). The systems were modeled using the OPLS4 force field. Prior to the production runs, all simulation systems underwent Desmond’s default eight-stage relaxation protocol. Pressure and temperature were maintained at 1 atm and 300 K, respectively, using the isotropic Martyna–Tobias–Klein barostat and the Nose–Hoover thermostat (Sharanya et al., 2023). Long-range coulombic interactions were evaluated using the smooth particle mesh Ewald (PME) method, with a short-range cutoff was set at 9.0 Å (Antony and Vijayan, 2021). A time-reversible reference system propagator algorithm (RESPA) integrator was used, with an inner time step of 2.0 fs and an outer time step of 6.0 fs (Humphrey et al., 1996). Clustering algorithms were applied to the MD trajectory data to identify representative structures and reduce the dimensionality of the dataset for subsequent analyses (Shao et al., 2007).

### 2.6 Identification of potential human kinase substrates and their interaction with NiV proteins

We compiled human-viral protein-protein interactions (PPIs) data for NiV proteins from multiple databases to evaluate potential interactions between NiV proteins and kinase substrates. The data were retrieved from HVIDB downloaded on 1 December 2024 (Yang et al., 2021), HVPPI downloaded on 19 September 2024 (Li et al., 2022), VirHostNet downloaded on 15 November 2024 (Guirimand et al., 2015), IntAct downloaded on 31 October 2024 (Kerrien et al., 2012), and BIOGRID downloaded on 9 September 2024 (Oughtred et al., 2021). In the present study, we utilized the kinase-substrate data generated by Sugiyama et al. (2019), who used a high-throughput quantitative proteomics approach to profile 385 human kinases and identified 175,574 potential kinase substrates. Johnson et al. (2023) reported that approximately 90,000 serine and threonine phosphorylation sites have been identified. In their study, synthetic peptide libraries were used to profile the substrate sequence specificity of 303 Ser/Thr kinases. Similarly, Yaron-Barir et al. (2024) employed combinatorial peptide arrays to characterize the substrate sequence specificity of all human tyrosine kinases. Their findings demonstrated substantial diversity in the optimal residue patterns surrounding phosphorylation sites, providing insights into the functional organization of the human tyrosine kinome based on substrate motif preferences. In the present study, we applied a 90th-percentile cutoff to the kinase-substrate data from Johnson et al. (2023) and Yaron-Barir et al. (2024). Subsequently, we integrated all three datasets Johnson et al. (2023), Yaron-Barir et al. (2024), and Sugiyama et al. (2019) to identify potential human kinase-substrate interactions with NiV proteins. The interaction network was constructed and visualized using Cytoscape, an open-source software platform for the integration and visualization of biomolecular interaction networks (Shannon et al., 2003).

## 3 Results

### 3.1 Kinase substrate phosphomotif analysis of NiV proteins as substrates for human protein kinase

**FIGURE 1:**
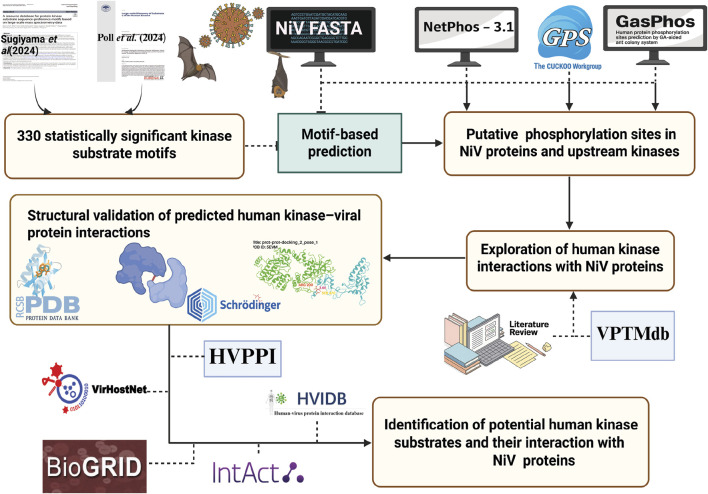
The workflow of the study.

A total of 377 NiV protein sequences, spanning a broad range of strains and isolates, were retrieved from the UniProt database. These sequences were sorted based on their NCBI and UniProt accessions (https://www.uniprot.org/ and https://www.ncbi.nlm.nih.gov/). Sequence alignments generated with Clustal Omega were then used to categorize the 377 sequences into nine distinct NiV proteins. By analyzing human protein kinome substrate motif patterns across these sequences, 51 human protein kinases were identified as being associated with 1180 putative phosphorylation sites distributed among the nine viral proteins. Of these, 24 were identified as serine/threonine kinases, 22 as tyrosine kinases, and 5 as dual-specificity kinases. The workflow of the study is given as Figure 1 and the data is provided in Supplementary Table S1.

Protein phosphorylation sites and their corresponding kinases in NiV proteins were predicted using GasPhos, NetPhos, and GPS tools. The predicted kinase-viral protein sites were subsequently compared with the kinase-substrate motif results identified above using the data from Poll et al. (2024). This integrated approach revealed both overlapping and distinct kinase predictions for each phosphorylation site. Kinase prediction analysis revealed that phosphorylation sites are distributed across multiple structural proteins of NiV, including the N protein, P protein, W protein, Fusion glycoprotein (F0), Glycoprotein, and C protein. Using a combination of computational tools and kinase substrate motif-based approaches, a total of 26 unique phosphorylation sites were identified as common predictions across multiple approaches. The NiV-P and W proteins exhibited the highest number of predicted 8 phosphorylation sites, followed by the Nucleoprotein with 6 sites. In contrast, the Glycoprotein and Fusion glycoprotein F0 showed 2 predicted sites each, while the C Protein had a single predicted phosphorylation site.

**FIGURE 2:**
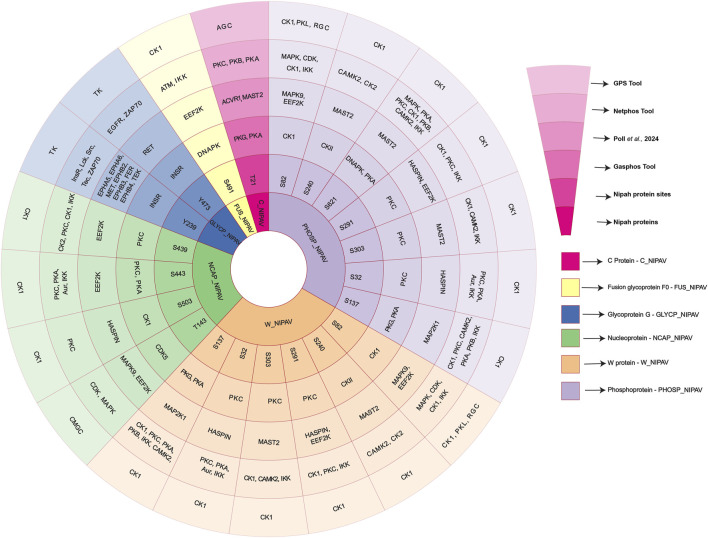
This figure illustrates the common NiV protein phosphorylation sites predicted by human kinase substrate motif analysis (Poll et al., 2024) and computational tools (GPS 5.0, NetPhos, and GasPhos), which also identified the corresponding kinases.

Kinase-substrate motif-based predictions derived from the study by Poll et al. (2024), are kinase-specific, enabling the identification of individual kinases like MAPK9 and EEF2K based on kinase substrate preferences (Poll et al., 2024). In contrast, computational tools such as GPS 5.0, NetPhos, and GasPhos generally predict the kinases based on the family, like MAPK, Cyclin-dependent kinase (CDK), Casein kinase 1 (CK1), or CMGC. For example, at the T143 site of the Nucleoprotein, MAPK9 was identified through motif-based prediction (Poll et al., 2024), while GPS 5.0 broadly assigned the site to the CMGC group, and NetPhos predicted MAPK and CDK family kinases. GasPhos further suggested CDK5, reflecting a partial overlap at the subfamily level. Similarly, at the S82 site in the Phosphoprotein and W Protein, motif-based analysis indicated MAPK9 and EEF2K as likely regulators. However, tool-based predictions highlighted kinase families such as MAPK, CDK, CK1, and Inhibitor of κB Kinase (IKK), with GPS classifying them again under the CMGC or RGC families. The results are presented in Figure 2 and Supplementary Table S2.

### 3.2 Characterization of phosphosites in protein kinases altered by NiV infection

We evaluated the 51 protein kinases and their phosphorylation sites identified through kinase substrate motif analysis and investigated the phosphorylation sites in NiV reported in the literature. Experimentally confirmed or validated phosphorylation sites from various NiV proteins were compiled from several published studies (Shiell et al., 2003; Huang et al., 2011; Edwards et al., 2020). Through our dataset, we identified S451 of the NiV-N protein as a phosphorylation site previously confirmed by experiments, as reported in VPTMdb (Xiang et al., 2021; Huang et al., 2011). Using kinase substrate motif analysis, we further predicted the human protein kinases LRRK2 and HASPIN as potential upstream regulators of this site. This highlights S451 as a key phosphosite in the nucleocapsid protein, supported by both experimental validation and motif-based kinase prediction. Based on previous studies, although the exact phosphorylation sites on paramyxoviral proteins may vary, the cellular kinases responsible for phosphorylation are generally conserved across the family (Shiell et al., 2003). Studies have demonstrated that mitogen-activated protein kinases, cyclin-dependent kinase 5, and glycogen synthase kinase 3 play vital roles in intracellular signaling and may be involved in the phosphorylation of P proteins within the *Paramyxoviridae* family (Pelech, 1995). In our analysis, we identified two phosphorylation sites, T410 and T438, along with their upstream kinases, located within the conserved region of the NiV-W protein. The upstream human kinase EEF2K was predicted to phosphorylate both sites, whereas MAPK9, another upstream human kinase, was specifically predicted to phosphorylate the site T438. These findings align with previously reported conserved phosphorylation sites within the distinct C-terminal region of the NiV-W protein (residues 408–450), a region critical for its interactions with the 14-3-3 protein family (Edwards et al., 2020). Previous studies have reported phosphorylation of the NiV-P protein at residues S240 and S472 (Shiell et al., 2003). kinase-substrate motif analysis predicted HASPIN as the upstream kinase for S240 and MAST2 as the upstream kinase for T239 and S472.

### 3.3 Identification of key human kinases through phosphosite frequency mapping in NiV proteins

**FIGURE 3:**
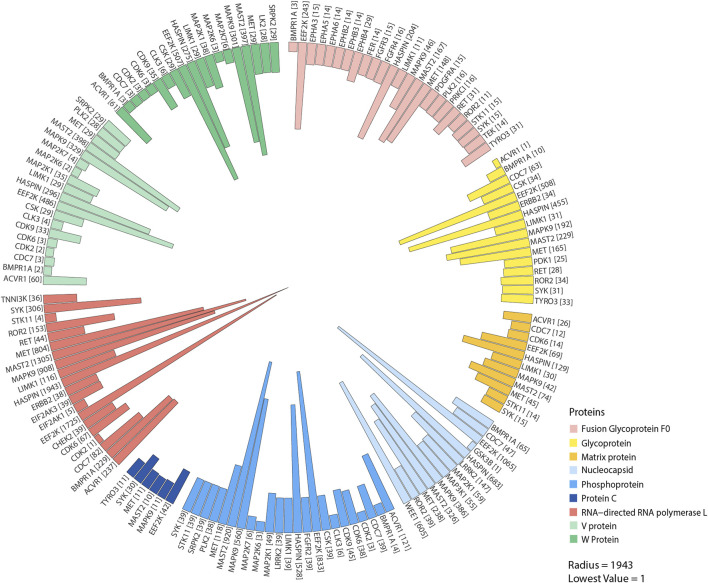
This circular stacked bar plot illustrates the kinases identified through human kinase substrate motif analysis that potentially phosphorylate multiple NiV proteins at multiple sites based on their frequency of occurrence.

A redundancy-based analysis of phosphorylation sites across different NiV proteins and kinases was performed using the results obtained from the kinase-substrate motif–based analysis. This analysis revealed common host kinases capable of phosphorylating multiple sites in NiV proteins across various NiV variants. This analysis highlights the pivotal roles of several key kinases, including EEF2K, HASPIN, MAST2, MAPK9, Hepatocyte growth factor receptor [MET], Tyrosine-protein kinase receptor [TYRO3], Proto-oncogene tyrosine-protein kinase receptor Ret [RET], Ephrin type-B receptor 4 [EPHB4], Serine/threonine-protein kinase PLK2 [PLK2], and Fibroblast growth factor receptor 4 [FGFR4], which are predicted to phosphorylate these viral proteins and may play critical roles in their regulation. These kinases play crucial roles in phosphorylating key NiV proteins such as RNA-directed RNA polymerase L, nucleocapsid, p, glycoprotein, fusion glycoprotein F0, V protein, and W protein. These host kinase-NiV protein interactions are likely to play a pivotal role in NiV infection, modulating viral replication and disease progression. Supplementary Figure S1 depicts a circular stacked bar plot from the redundancy-based phosphosite analysis, showing the prevalence of kinases predicted to phosphorylate NiV viral proteins (See Supplementary Figure S1). The Sunburst diagram illustrates the frequency of viral proteins and their respective phosphorylation sites (as shown in Figure 3; Supplementary Figure S1).

### 3.4 Molecular docking results of kinases and their NiV protein sites

Protein–protein docking analysis was performed to evaluate the binding affinities and stability of interactions between the predicted phosphorylation sites on NiV proteins and their corresponding human kinases EEF2K, HASPIN, and MAPK9. Docking using BioLuminate in Schrodinger Maestro 2025-3 generated 30 poses for each interaction, and the best pose was selected based on PIPER cluster size and PIPER pose energy (Navhaya et al., 2024). The most stable kinase NiV protein interactions were then analyzed further, revealing distinct and proteome specific phosphorylation profiles across NiV variants. The human protein kinase EEF2K displayed the most significant interaction with the NiV glycoprotein (PDB ID: 8FNY_7TXZ), which was characterized by a cluster size of 59 and a PIPER pose energy of −2081.769 kcal/mol. In addition, EEF2K showed a strong binding affinity with the NiV-P (PDB ID: 8FNY_9FUX), with a cluster size of 73, and a PIPER pose energy of −1838.156 kcal/mol, making it the second most favourable interaction. The NiV fusion glycoprotein (PDB ID: 8FNY_5EVM) has a cluster size of 79 and a PIPER pose energy of −1046.022 kcal/mol. In contrast, the NiV-N (PDB ID: 8FNY_7NT5), which had a cluster size of 55, and a PIPER pos energy of −854.964 kcal/mol (Supplementary Table S3). The relationship between human kinase EEF2K and the NiV proteins including fusion glycoprotein, glycoprotein, N protein, and P protein has not been reported before, suggesting that these proteins may play a role in promoting NiV infection and spread throughout the human body.

Results revealed interactions between kinase and viral protein have shown that the human protein kinase HASPIN and the NiV proteins have distinct binding properties and phosphorylation-related changes. HASPIN and RNA-dependent RNA polymerase (PDB ID: 3DLZ_9IR4) interaction had the PIPER pose energy of −2044.791 kcal/mol, and a cluster size of 48. HASPIN_Nucleocapsid (PDB ID: 3DLZ_7NT5) had the lowest PIPER pose energy (−1668.38 kcal/mol) and a cluster size of 55. The HASPIN and Glycoprotein protein complex (PDB ID: 3DLZ_7TXZ) complex showed interactions with a PIPER pose energy of −1565.627 kcal/mol and a cluster size of 68. The interaction with the highest docking scores among them was HASPIN and Matrix Protein complex (PDB ID: 3DLZ_7SKT). Its highest cluster size was 127, and the PIPER pose energy was −1463.435 kcal/mol. Finally, with a cluster size of 38, a PIPER pose energy of −1392.027 kcal/mol, and a HASPIN-Fusion Glycoprotein complex (PDB ID: 3DLZ_5EVM) demonstrated a moderate binding affinity. These findings emphasize the differences in phosphorylation-mediated interactions between HASPIN proteins, providing insight into their structural and functional relevance. HASPIN exhibited the most extensive phosphorylation profile with RNA-dependent RNA polymerase, Fusion glycoprotein, Matrix protein, and Nucleocapsid proteins. These kinases could be critical in phosphorylating NiV proteins during the infection process (Supplementary Table S3).

Our analysis indicates that the human protein kinase MAPK9 might phosphorylate the NiV Matrix Protein (PDB ID: 7N8T_7SKT), as demonstrated by a strong interaction with a cluster size of 74, and a PIPER pose energy of −776.583 kcal/mol. This significant connection suggests that the NiV matrix protein, responsible for viral entry, is regulated by the human protein kinase MAPK9. The hypothesis that MAPK9 phosphorylates the NiV-P (PDB ID: 7N8T_9IR4) is further supported by a cluster size of 161, and a PIPER pose energy of −1205.792 kcal/mol. Considering the essential role of the Phosphoprotein in the transcription and replication of viral RNA, along with its capability to influence host immune responses, phosphorylation by MAPK9 could have significant consequences for the pathogenicity of the NiV (Supplementary Table S3).

To compare protein–protein docking results obtained using BioLuminate in Schrodinger Maestro 2025-3, we performed HADDOCK web-based server for docking of the EEF2K–NiV Fusion Glycoprotein F0 complex using HADDOCK, which generated 10 clusters. These clusters were evaluated based on HADDOCK score, Z-score, RMSD, van der Waals energy, electrostatic energy, desolvation energy, restraint violation energy, and buried surface area. Cluster 1 emerged as the most reliable solution, with the lowest HADDOCK score (74.8 ± 9.9), the most negative Z-score (−2.1), low RMSD (1.5 ± 1.4 Å), strong electrostatic interactions (−451.8 ± 85.7 kcal/mol), and a substantial buried surface area (2252.1 ± 47.2 Å^2^), indicating a stable and well-converged interaction interface. Clusters 8–10 showed higher HADDOCK scores (90.9–95.8) and less negative Z-scores (−0.7 to −0.3), reflecting moderate reliability, while clusters 4–6 exhibited the highest scores (110.8–119.0) and positive Z-scores (0.4–1.8), indicating lower confidence. Interestingly, some clusters, such as Cluster 8, displayed large buried surface areas (2827.6 ± 199.8 Å^2^) despite lower reliability, suggesting extensive interaction surfaces (Supplementary Table S4).

Docking with BioLuminate in Schrodinger Maestro 2025-3 produced a larger top-ranked cluster (79 poses) than HADDOCK Cluster 1 (34 poses). This greater convergence indicates that BioLuminate in Schrodinger Maestro 2025-3 offers a more reliable prediction of the EEF2K–NiV fusion glycoprotein f0 interaction compared to web-based server HADDOCK.

### 3.5 Physicochemical characterization of identified kinases

The physicochemical characteristics of the identified kinases were determined using the MPP Profiler tool to gain insights into their structural and functional attributes. The analysis revealed differences in molecular weight, isoelectric point, aliphatic index, instability index, and hydropathicity. For instance, EEF2K (length 725 aa) exhibited a molecular weight of 82.1 kDa, a low isoelectric point (pI 5.16), and a negative charge at physiological pH, suggesting an overall acidic nature and instability (instability index 42.69). In contrast, MAPK9 (length 424 aa, MW 48.1 kDa, pI 5.41) also showed instability, while HASPIN (length 798 aa, MW 88.5 kDa) displayed a higher pI (9.32) and a positive charge at physiological pH, indicating a basic and unstable profile. A complete list of physicochemical properties for all kinases is provided in Supplementary Table S5.

### 3.6 Protein- protein interaction analysis of EEF2K and viral proteins

Protein–protein interactions between kinases and NiV proteins from the best docking pose were analyzed using the protein interaction analysis module in BioLuminate in Schrodinger Maestro 2025-3 (Navhaya et al., 2024).

#### 3.6.1 Human kinase EEF2K with NiV fusion glycoprotein F0

The human protein kinase EEF2K and NiV fusion glycoprotein complex (PDB ID: 8FNY, 5EVM) exhibits strong molecular interactions, particularly involving S466 and S470 on the NiV fusion glycoprotein, which interact with key residues in the kinase domain of EEF2K. The human kinase EEF2K interacts with the NiV fusion glycoprotein at phosphosite S466, forming a hydrogen bond with L193 and R200 within the kinase domain, while also exhibiting three van der Waals clashes with E196 and two clashes with R200 in the complex. The high surface complementarity score of 0.76% and 100.00% buried solvent-accessible surface area (SASA) further supports the stability of this interaction. With a surface complementarity score of 0.63% and 92.40% buried SASA, NiV fusion glycoprotein at phosphosite S470 also interacts with E197 in the kinase domain of EEF2K through two van der Waals collisions and one hydrogen bond, suggesting a major role in complex stability.

#### 3.6.2 Human kinase EEF2K with NiV nucleocapsid

The interaction analysis between the human kinase EEF2K and the NiV-N protein revealed that the phosphosite S289 of the NiV-N protein interacts with residue F155 within the kinase domain of EEF2K at a distance of 4.0 Å, indicating close spatial proximity between EEF2K and the NiV-N protein (PDB IDs: 8FNY, 7NT5). The buried SASA of 35.40% and a surface complementarity score of 0.33 suggest that this interaction contributes moderately to the overall stability of the complex.

#### 3.6.3 Human kinase EEF2K with NiV phosphoprotein

The interaction analysis of the EEF2K and NiV-P protein complex (PDB ID: 8FNY–9FUX) reveals that the NiV Phosphoprotein phosphosite at S650 interacts with multiple residues within the kinase domain, including H230, S165, Y167, and N166, at distances ranging from 1.4 Å to 3.6 Å. Notably, NiV-P protein phosphosite at S650 shows van der Waals clashes, with 20 with H230, 14 with S165, and 7 with Y167 within the EEF2K kinase domain, indicating a strong steric influence in the interaction. With a surface complementarity score of 0.02% and 94.80% buried SASA, this interaction seems to have a significant structural function. Furthermore, S165, Y167, and H230 of EEF2K are involved in multiple clashes with the S650 residue. As a phosphorylation site, S650 of the NiV-P protein may serve as a critical regulatory target of EEF2K, potentially modulating phosphoprotein function and influencing viral regulatory mechanisms.

#### 3.6.4 Human kinase HASPIN with NiV fusion glycoprotein

**FIGURE 4:**
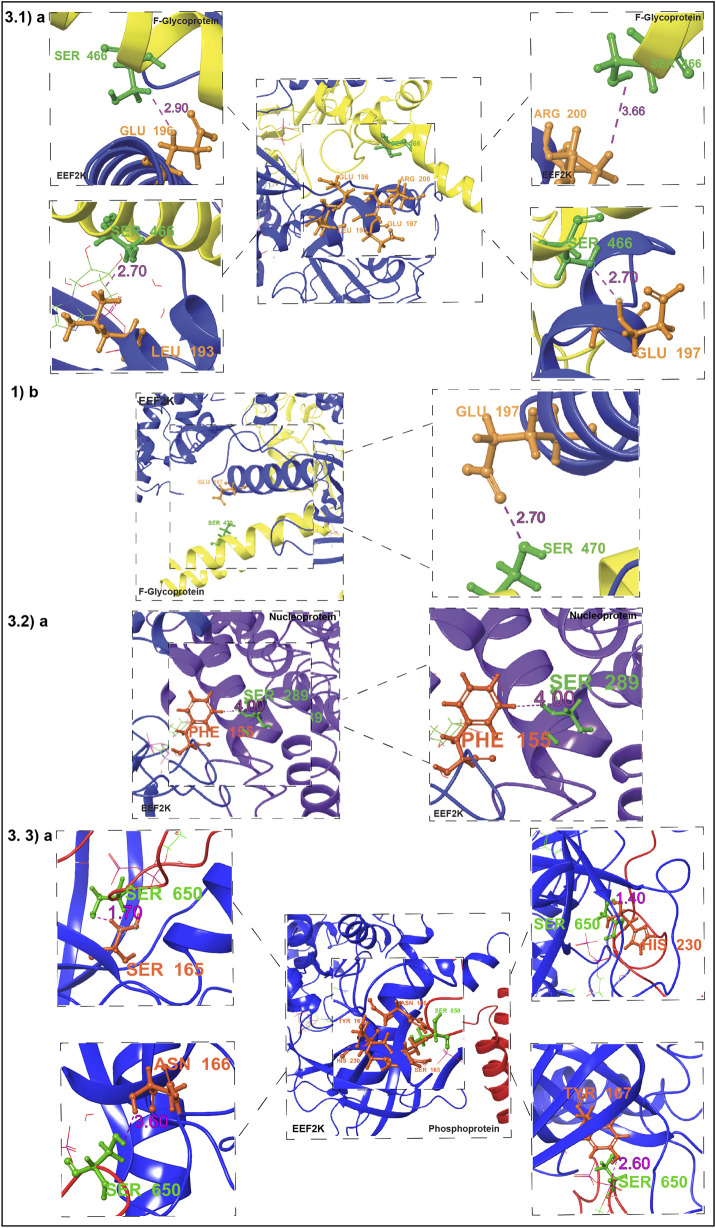
Protein-Protein Docking and Identification of Kinase Substrate Phosphorylation Sites in NiV Proteins. This figure illustrates the docking interactions between host kinases and NiV proteins, highlighting key kinase-substrate phosphorylation sites identified in the NiV protein sequences. 4.1) (a–d) Interaction between EEF2K and the NiV Fusion Glycoprotein. In the figure, yellow indicates NiV proteins, and blue indicates kinases. 4.2) a. Interaction between EEF2K and the NiV P protein. In the figure, violet indicates NiV P protein and blue indicates kinases. 4.3) a. Interaction between EEF2K and the NiV nucleocapsid protein. In the figure, violet indicates nucleocapsid and blue indicates kinases.

The interactions between human kinase HASPIN and the NiV fusion glycoprotein complex, residues S261 and T321 of the NiV Fusion Glycoprotein exhibit low surface complementarity (0.04 and 0.11, respectively) and minimal buried SASA (25.40% and 10.10%), with human kinase HASPIN. Table 1 Lists the key residues involved in the interactions between the kinases and NiV proteins (Supplementary Table S3; Figures 4A,B).

**TABLE 1:** Summary of key interacting residues derived from protein–protein interaction analysis between kinases and viral proteins.

Protien Name	PDB ID	Residue	Closest	Distance	Specific Interactions	# HB	# Salt Bridges	# Pi Stacking	# Disulfides	# vdW Clash	Surface Complementarity	Buried SASA
EEF2K_FUSION GLYCOPROTEIN	8FNY_5EVM	B:404:Ser				0	0	0	0	0	0.09	0.012
EEF2K_FUSION GLYCOPROTEIN	8FNY_5EVM	B:408:Thr				0	0	0	0	0	0	0.283
EEF2K_FUSION GLYCOPROTEIN	8FNY_5EVM	B:466:Ser	C:200:Arg C:193:Leu C:197:Glu C:196:Glu	2.6 A 2.7 A 2.7 A 2.9 A	1x hb to C:193:Leu 3x clash to C:196:Glu 1x hb, 2x clash to C:200:Arg	2	0	0	0	5	0.76	1
EEF2K_FUSION GLYCOPROTEIN	8FNY_5EVM	B:470:Ser	C:197:Glu C:200:Arg	2.7 A 2.8 A	1x hb, 2x clash to C:197:Glu 2x clash to C:200:Arg	1	0	0	0	4	0.71	0.836
EEF2K_ NUCLEOCAPSID	8FNY_7NT5	B:289:Ser	C:155:Phe	4.0 A		0	0	0	0	0	0.33	0.354
EEF2K_PHOSPHOPROTEIN	8FNY_9FUX	B:650:Ser	C:230:His C:165:Ser C:167:Tyr C:166:Asn	1.4 A 1.7 A 2.6 A 3.6 A	14x clash to C:165:Ser 7x clash to C:167:Tyr 20x clash to C:230:His	0	0	0	0	41	0.02	0.948
EEF2K_PHOSPHOPROTEIN	8FNY_9FUX	C:165:Ser	B:650:Ser B:649:Ala	1.7 A 1.7 A	6x clash to B:649:Ala 14x clash to B:650:Ser	0	0	0	0	20	-0.16	0.741
EEF2K_PHOSPHOPROTEIN	8FNY_9FUX	C:166:Asn	B:650:Ser	3.6 A		0	0	0	0	0	0.16	0.422
EEF2K_PHOSPHOPROTEIN	8FNY_9FUX	C:167:Tyr	B:650:Ser	2.6 A	7x clash to B:650:Ser	0	0	0	0	7	0.31	0.458
EEF2K_PHOSPHOPROTEIN	8FNY_9FUX	C:230:His	B:650:Ser B:644:Phe B:651:Gln B:649:Ala	1.4 A 2.2 A 3.1 A 3.4 A	16x clash to B:644:Phe 20x clash to B:650:Ser	0	0	0	0	36	0.24	0.85

NB: A and C is the chains of EEF2K and B is the chain of NiV proteins.

Cluster 1 of the HADDOCK docking results exhibited the most favorable interaction profile, with the lowest HADDOCK score (74.8 ± 9.9) and the most negative Z-score (−2.1), indicating high reliability. It showed a low RMSD (1.5 ± 1.4 Å), strong electrostatic energy (−451.8 ± 85.7 kcal/mol), and a substantial buried surface area (2252.1 ± 47.2 Å^2^), reflecting a stable and well-converged docking solution. However, since HADDOCK lacks the capability to analyze site-specific interactions between kinases and NiV viral proteins, this limitation was addressed using the Protein Interaction Analysis module in BioLuminate in Schrodinger Maestro 2025-3 (Supplementary Table S4).

We integrated data from protein phosphorylation site prediction tools, which are used to predict host kinases and their corresponding phosphosites. Importantly, some of these predicted phosphosites were also identified in the protein–protein interaction data of NiV host interactions, further supporting their potential biological relevance. These potential phosphorylation sites, which may undergo phosphorylation during infection, include S481 on the NiV-P protein, S466, S470, and S404 on the NiV Fusion Glycoprotein F0, and S289 on the NiV-N protein (Supplementary Table S6).

### 3.7 Molecular dynamics analysis of the human protein kinase EEF2K–NiV fusion glycoprotein complex

Trajectory maps are user-friendly simulation analysis tools that provide results that are easy to interpret (Kozic and Bertosa, 2024). We analyzed the MD simulation results over a 300-nanosecond trajectory, examining interaction patterns across different frames. Trajectory maps were generated from trajectories containing 1 to 3,000 frames, with every 300 frames representing a single frame in the map, resulting in 10 frames per replicate for all three replicates. Notably, the residue pairs C:R252–B:D304 and C:K205–B:D304 (C chain represents the human kinase EEF2K, and the B chain corresponds to the Fusion Glycoprotein) were repeatedly observed across multiple frames and replicates, indicating their stable and persistent involvement in the interaction interface. Here, R252 and K205 are residues located within the human kinase domain of EEF2K, while D304 belongs to the Fusion Glycoprotein. These interactions predominantly involved hydrogen bonds and salt bridges, often with 100% pose occupancy, highlighting their structural stability during the entire simulation period. The persistence of these contacts suggests that D304 on the Fusion Glycoprotein forms a stable electrostatic and hydrogen bonding network with key residues of EEF2K, likely playing an important role in substrate recognition and positioning within the kinase interface (Supplementary Table S7).

We analyzed the protein–protein interaction profiles obtained from replicate 1 and replicate 3 of the MD simulations to evaluate the stability of predicted kinase-substrate interaction sites over a 300 ns trajectory. Notably, the residue pairs R200- S466, K202–S466, and R200–S470 were consistently observed across multiple frames in both replicates. Although these interactions did not consistently exhibit hydrogen bonds or salt bridges throughout the trajectory, their recurrent appearance across different frames indicates that these predicted phosphorylation site regions (S466 and S470) remain in close spatial proximity to key kinase residues (R200 and K202). This suggests the presence of transient or weak interactions that may assist in substrate recognition or alignment during the docking and phosphorylation process. A hydrogen bond observed between R200 and S470 in the final frame (3000 frames) of replicate 3 further supports the potential functional relevance of this interaction within the kinase-substrate interface (Supplementary Table S8).

**FIGURE 5:**
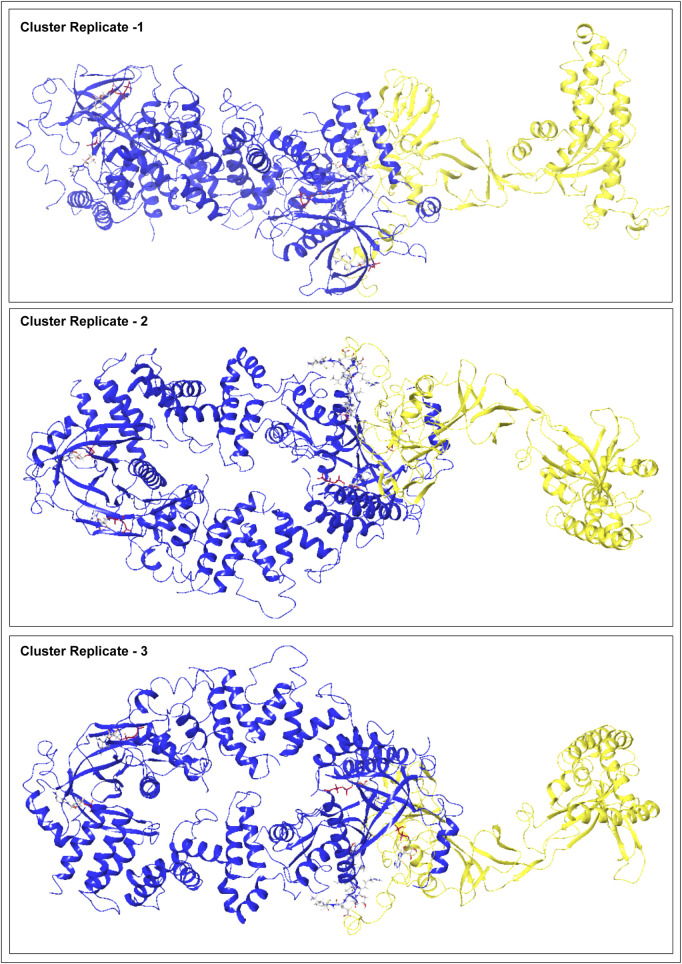
Complex structure of EEF2K (blue) and the NiV Fusion Glycoprotein (yellow) after clustering analysis. Representative conformation obtained from clustering of the molecular dynamics (MD) simulation trajectories.

**FIGURE 6:**
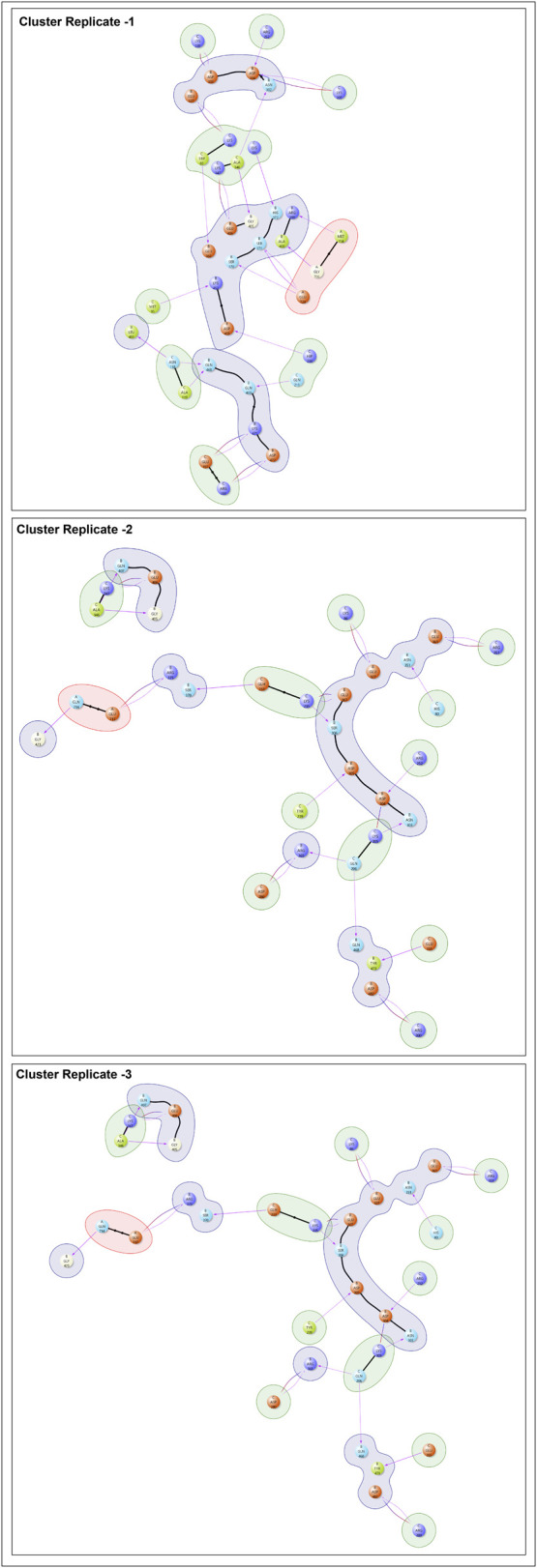
Interaction analysis of the EEF2K–Fusion Glycoprotein complex after cluster analysis. This figure highlights the key residue–residue interactions between EEF2K and the NiV Fusion Glycoprotein.

After performing clustering analysis on the MD trajectories of the complex across three replicates, several residue interactions were consistently maintained (Figures 5). In particular, the interactions C: R252–B: D304, C: K205–B: D304/B: N303, and C: K86–B: E327 were observed across all cluster representatives. These interactions exhibited short distances of 1.6–1.7 Å, reflecting tight contacts, and involved both hydrogen bonds and salt bridges, highlighting strong electrostatic and polar contributions to interface stability (Supplementary Table S9). Importantly, the same interactions were also consistently observed in the raw MD trajectories across multiple frames and replicates, further confirming their stability and persistent involvement in the protein–protein interface (Figures 6).

### 3.8 EEF2K substrates are known interactors of NiV proteins

**FIGURE 7:**
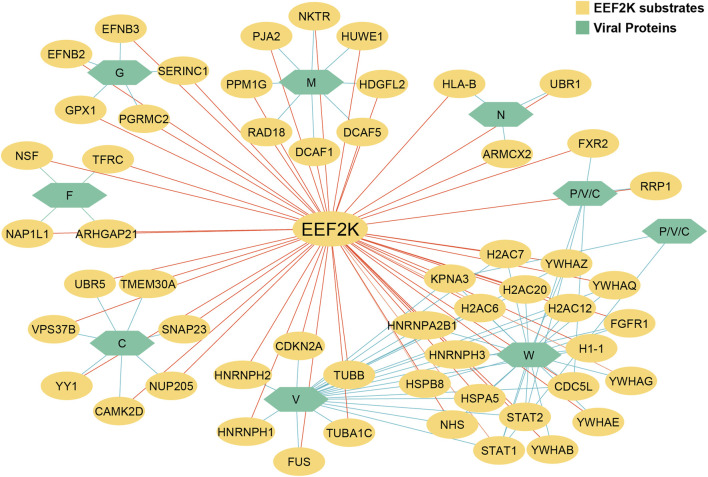
This figure represents the network of EEF2K substrate proteins, illustrating their interactions with NiV proteins based on known human–viral protein–protein interaction data.

Using human–viral protein–protein interaction (PPI) network data from databases and kinase-specific substrate data analyzed from Sugiyama et al. (2019), Johnson et al. (2023), and Yaron-Barir et al. (2024), a total of 56 EEF2K substrates were identified to interact with seven NiV proteins (Sugiyama et al., 2019; Johnson et al., 2023; Yaron-Barir et al., 2024). Non-structural protein V (V_NIPAV) interacts with 17 substrates of human kinase substrates. The NiV Phosphoprotein (PHOSP_NIPAV) was associated with two substrates, including FXR2, while Glycoprotein G (GLYCP_NIPAV) shows interactions with eight substrates, such as HLA-B, whereas the Fusion Glycoprotein F0 (FUS_NIPAV) has four substrates, including TFRC, whereas Protein C (C_NIPAV) engaged with seven substrates, such as CAMK2D. The Matrix Protein (MATRX_NIPAV) and Protein W (W_NIPAV) were found to interact with seven and eight substrates, respectively, among which are DCAF1 and FGFR1. These interactions between NiV viral proteins and EEF2K substrates may facilitate the hijacking of host cellular pathways, thereby enhancing viral survival, replication, and overall pathogenesis within the host (Figure 7).

### 3.9 Conserved phosphorylation sites in NiV proteins across strains and isolates

Our analysis of experimentally validated phosphorylation sites in NiV proteins reveals their high degree of conservation and suggests a critical role in viral function. The Phosphoprotein of NiV displays highly conserved phosphorylation sites across various strains and isolates, emphasizing their essential roles in viral activities. Notably, the phosphorylation site at T239, predicted to be modulated by the human protein kinase MAST2, is fully conserved across all analyzed strains and isolates. Likewise, the phosphorylation site located at S240, which is regulated by the human protein kinases HASPIN and MAST2, also exhibits 100% conservation across the strains and isolates of NiV. The N protein contains a phosphorylation site at S451, with human protein kinases LRRK2 and HASPIN recognized as possible upstream regulators. This site is conserved in 97.3% and 89.9% of strains and isolates, respectively. Furthermore, the human protein kinase EEF2K is predicted to phosphorylate the NiV W protein at T410 and T438, with conservation rates of 86.2% and 89.6%, respectively.

Our protein-protein docking analysis for the NiV fusion glycoprotein f0 has revealed critical phosphorylation sites that are highly conserved across different strains and isolates. The human protein kinase EEF2K is predicted to target seven phosphorylation sites on the NiV fusion glycoprotein f0 protein: S402, S404, T408, T346, S458, S466, and S470. Most of these sites are conserved in 93.75% of the analyzed strains and isolates, while S458 is conserved in 87.5%. Additionally, the human kinase HASPIN is predicted to phosphorylate two conserved sites, T321 and S261, both of which are conserved in 93.75% of the strains.

**FIGURE 8:**
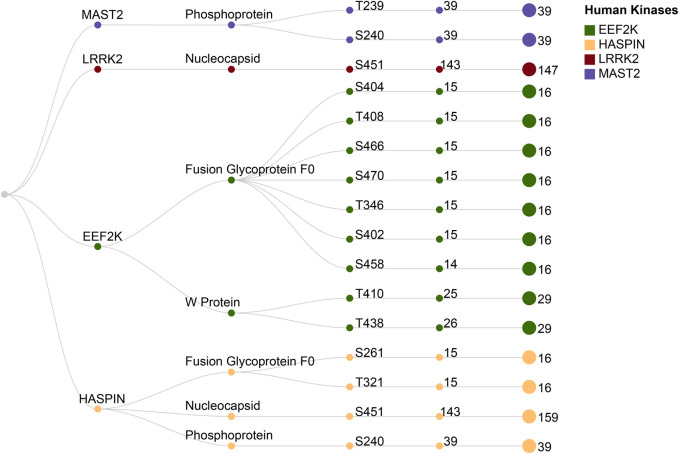
The figure illustrates the conservation of experimentally validated phosphorylation sites in NiV proteins and their corresponding upstream human kinases, predicted through kinase-substrate motif analysis. These sites within each viral protein were analyzed and found to be conserved across different strains and isolates of the NiV.

The NiV fusion glycoprotein is essential for membrane fusion, which allows the NiV to penetrate cells (Weis and Maisner, 2015). Due to its critical role, the NiV fusion protein is considered a promising target for vaccine and treatment development. Protein-protein docking with Human protein kinases EEF2K and HASPIN revealed interaction sites in the NiV F protein, indicating potential phosphorylation pathways that could facilitate the virus’s entrance into host cells. The high conservation of these phosphorylation motifs highlights their importance in NiV biology, emphasizing their potential roles in interactions between the host and the pathogen. These findings provide valuable insights into virus–host dynamics and suggest that targeting the human kinases involved in these phosphorylation events could represent a promising antiviral strategy (Figure 8; Supplementary Table S10).

## 4 Discussion

Human protein kinases are critical in regulating host-pathogen interactions, but their significance in NiV infections has not been well explored. Recently, we employed molecular docking and dynamics studies to identify potential inhibitors of the NiV glycoprotein from Indian medicinal plants (Abhinand et al., 2023) and further adopted a deep learning and molecular modeling approach to explore the repurposing potential of Cangrelor as an inhibitor of NiV (John et al., 2025). Identifying and comprehending host-virus interactions is critical for understanding infection mechanisms and applying this knowledge to the development of vaccines or antiviral therapies. Historically, many approaches have been employed to identify protein-protein interactions (PPIs) between hosts and viruses. The viral polymerase complex of NiV, consisting of the polymerase (L) and the phosphoprotein, is responsible for replicating and transcribing the viral RNA genome (Yang et al., 2024). NiV employs multiple strategies to evade the host immune response by targeting the STAT1 pathway. While the P and V proteins retain STAT1 in the cytoplasm, preventing its nuclear translocation, the W protein actively sequesters STAT1 within the nucleus. This complementary mechanism establishes both cytoplasmic and nuclear barriers that block STAT1 mediated transcription of antiviral genes, thereby undermining the host’s ability to mount an effective IFN-I response. These findings suggest that the NiV P, V, and W proteins work together to suppress IFN-I signaling through their interactions with STAT1 (Shaw et al., 2004; Rodriguez et al., 2002). The M protein of the NiV inhibits the activation of type I interferon by interfering with the TRIM6-IKKε pathway. This disruption hinders the creation of K48-linked polyubiquitin chains, which are vital for antiviral signaling. By obstructing this crucial immune response, NiV-M facilitates viral immune evasion, thereby promoting effective replication and infection (Bharaj et al., 2016). NiV uses essential glycoproteins to adhere and fuse virions to the host cell membrane, which is vital for its infectivity (Lamb et al., 2007; Weis and Maisner, 2015; Byrne et al., 2023). The proteolytic activation of the fusion (NiV-F) protein is crucial for NiV entry and replication. This activation is an essential prerequisite for viral infectivity, enabling membrane fusion and facilitating the spread of the virus. Glycoprotein G interacts with the host receptors ephrin-B2 and ephrin-B3, which facilitates viral entry into the host cells (Negrete et al., 2006; Negrete et al., 2005; Moll et al., 2004). The nucleocapsid protein is a critical structural component that envelopes the RNA genome, ensuring stability while regulating transcription and replication for effective viral propagation (Eshaghi et al., 2005). The helical nucleocapsid structure preserves the genome and serves as a template for viral RNA-dependent RNA polymerase (RdRp) activity (Chua et al., 2000; Ogino and Green, 2019).

The kinase-substrate phosphomotif pattern analysis revealed several kinases, including EEF2K, HASPIN, MAPK9, MAST2, and Spleen tyrosine kinase (SYK), that can phosphorylate multiple NiV proteins at various sites, suggesting regulatory roles during infection. EEF2K is a unique kinase that stimulates protein synthesis by phosphorylating and inhibiting eEF2. Because elongation is an energy-intensive process, EEF2K activity is regulated by multiple signaling pathways; changes in cellular energy might influence the expression of particular proteins (Liu and Proud, 2016). Rottlerin, an inhibitor of EEF2K, prevents eEF-2 phosphorylation and affects other kinases at lower concentrations, while at higher concentrations it reduces NiV replication by modulating PKC and EEF2K pathways, potentially inhibiting protein synthesis and regulating cellular energy homeostasis (Gschwendt et al., 1994; Wang et al., 2017; Aljofan et al., 2010). Huang et al. (2011) showed that NiV-N phosphorylation is critical in viral transcription and replication. By inhibiting NiV-N dephosphorylation with okadaic acid (OKA), they were able to identify its phosphorylation site at NiV-N S451, which was identified using peptide mass fingerprinting and mass spectrometry (Huang et al., 2011). HASPIN, a human kinase that phosphorylates histone H3 at threonine 3 (H3T3ph) during mitosis to recruit the chromosomal passenger complex, is autoinhibited in interphase and activated by multisite phosphorylation (Nguyen et al., 2014; Ghenoiu et al., 2013). We propose that HASPIN kinase acts as an upstream regulator of the NiV-N protein, most likely by facilitating its phosphorylation at S451, which is required for NiV-N’s activity in viral replication and transcription. Treatment with OKA, an effective phosphatase inhibitor, could enhance the phosphorylation of HASPIN and NiV-N, facilitating further investigations into their phosphorylation mechanisms. Similarly, LRRK2 may affect NiV-N phosphorylation at S451. However, the lack of complete structural model for the NiV-N protein, as well as missing sequence residues, limited our ability to perform protein-protein docking studies to investigate these kinases’ interactions with NiV-N. This study combined motif search and protein–protein docking analyses to explore host kinase interactions with NiV proteins. Our protein–protein docking analysis indicates that EEF2K and HASPIN strongly interact with NiV fusion glycoprotein and RNA-dependent RNA polymerase, suggesting a potential regulatory role in viral replication. The robust binding of EEF2K to viral phosphoproteins may reflect its involvement in modulating viral protein synthesis, consistent with its role in translational elongation, while interaction of HASPIN with NiV proteins points out its possible role in NiV-N phosphorylation. Molecular dynamics simulations further support the stability and functional relevance of these interactions, highlighting key residues such as R200, K202, and D304 in EEF2K that maintain persistent contacts with S466 and S470 on the NiV Fusion Glycoprotein, likely facilitating substrate recognition and phosphorylation. Notably, kinases such as LRRK2, HASPIN, MAST2, and EEF2K are predicted to be upstream regulators of experimentally validated phosphorylation sites on NiV proteins, including S451 on NiV-N, S240 on NiV-P, and T410/T438 on NiV-W protein. These findings suggest that these kinases could play important roles in NiV replication and immune modulation, though further experimental validation is necessary to confirm their functional significance. Conservation analysis in this study was limited to NiV strains; future studies extending this analysis to other Henipaviruses, such as Hendra and Cedar virus, could provide broader evolutionary insights and generalization of the identified phosphosites and kinase interactions. These findings suggest that these kinases could play important roles in NiV replication and immune modulation, and future studies could experimentally validate these predictions using approaches such as site-directed mutagenesis, kinase assays, and phosphoproteomics.

## 5 Conclusion

The ongoing battle between humans and NiV drives rapid evolution on both sides. Despite advances in understanding viral genetics and developing new drugs, NiV outbreaks are becoming more frequent and economically burdensome, highlighting the importance of studying virus–host interactions. Our findings suggest that EEF2K, a host kinase that phosphorylates multiple NiV proteins including the fusion glycoprotein, nucleocapsid, and phosphoprotein may regulate infection by targeting key residues in these viral proteins. Targeting such kinases with specific inhibitors could disrupt viral functions and reduce disease severity. Considering the zoonotic nature of NiV, modulating host kinases could limit viral replication across species. By coupling kinase substrate phosphomotif analysis with *in silico* docking approaches, we identified human kinases linked to experimentally validated NiV phosphorylation sites, providing a framework for host-targeted antiviral strategies. Integrating human, animal, and environmental health efforts, including surveillance, early detection, public awareness, vaccine development, genomic monitoring, healthcare reinforcement, and global collaboration, offers a proactive One Health strategy for comprehensive NiV preparedness and response.

## Data Availability

The original contributions presented in the study are included in the article/Supplementary Material, further inquiries can be directed to the corresponding authors.

## Glossary

NiV: Nipah Virus
EEF2K: Eukaryotic elongation factor 2 kinase
HASPIN: Histone H3 associated Protein Kinase
MAPK9: Mitogen-activated protein kinase 9
MAST2: Microtubule-Associated Serine/Threonine Kinase 2
SYK: Spleen tyrosine kinase
CDK: Cyclin-dependent kinase
CK1: Casein kinase 1
IKK: Inhibitor of κB Kinase
TYRO3: Tyrosine-protein kinase receptor
MET: Hepatocyte growth factor receptor
TYRO3: Tyrosine-protein kinase receptor
RET: Proto-oncogene tyrosine-protein kinase receptor Ret
EPHB4: Ephrin type-B receptor 4
PLK2: Serine/threonine-protein kinase PLK2
FGFR4: Fibroblast growth factor receptor 4
ssRNA: negative-sense single-stranded RNA
N: Nucleocapsid
P: Phosphoprotein
M: Matrix
F: Fusion
G: Attachment
L: RNA-dependent RNA polymerase
VPTMdb: Viral post-translational modification database
FDA: Food and Drug Administration
S: Serine
T: Threonine
Y: Tyrosine
PPI: Protein-protein interaction
HVIDB: Human-Virus Interaction DataBase
HVPPI: Human–Virus Protein-Protein Interaction database
VirHostNet: Virus–Host Network
BIOGRID: Biological General Repository for Interaction Datasets
PDB: Protein Data Bank
STAT1: Signal transducer and activator of transcription 1
IFN-I: type-I interferons
NiV-M: NiV matrix protein
NiV-N: NiV nucleocapsid protein
H3T3ph: histone H3 at threonine 3
CPC: Chromosomal passenger complex
OKA: Okadaic acid
PKC: Protein kinase C
SASA: Solvent-accessible surface area
