# Supplementary figures and images for: Uncovering human kinase substrates in nipah proteome
Source: Front Bioinform. 2025 Dec 5;5:1678189. doi: 10.3389/fbinf.2025.1678189 (PMC12715814; doi:10.3389/fbinf.2025.1678189)

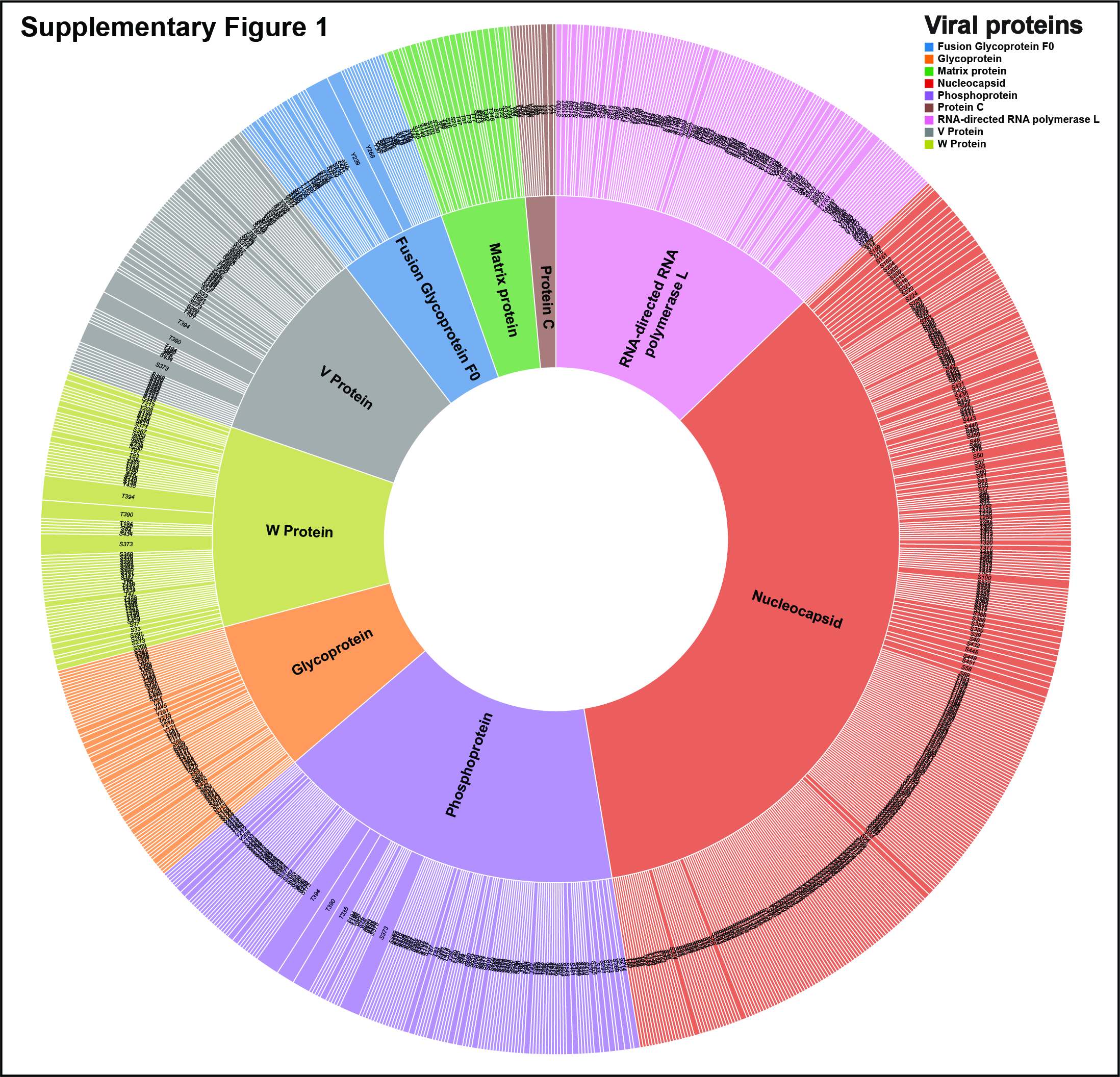

Supplement: Supplementary file 4 [file Image1.tif]
